# Synthesis of Al_2_O_3_–Fe_2_O_3_–FeAl_2_O_4_ Composites by Colloidal and Traditional Powder Routes of Nano-Al_2_O_3_–Fe_2_O_3_ Mixtures

**DOI:** 10.1021/acsomega.4c07928

**Published:** 2025-02-14

**Authors:** Cristian Cordova-Mayo, Daniel Fernández-González, Adolfo Fernández, Luis Felipe Verdeja, Alan Castillo-Rodríguez, Edén Amaral Rodríguez-Castellanos, Marina Hernández-Reséndiz, Linda García-Quiñonez, Cristian Gómez-Rodríguez

**Affiliations:** † Facultad de Ingeniería, Campus Coatzacoalcos, Universidad Veracruzana, Av. Universidad km 7.5 Col. Santa Isabel, Coatzacoalcos, Veracruz 96535, Mexico; ‡ Nanomaterials and Nanotechnology Research Center (CINN-CSIC), Universidad de Oviedo (UO), Principado de Asturias (PA), Avda. de la Vega, 4-6, El Entrego 33940, Spain; § Departamento de Ciencia de los Materiales e Ingeniería Metalúrgica, Escuela de Minas, Energía y Materiales, Universidad de Oviedo, Calle Independencia, s/n, Oviedo/Uviéu, Asturias 33004, Spain; ∥ Universidad Autónoma de Nuevo León, Av. Universidad S/N, San Nicolás de los Garza 66451, Mexico; ⊥ Centro de Investigación en Recursos Energéticos y Sustentables (CIRES), Universidad Veracruzana, Av. Universidad Veracruzana km 7.5, Col. Santa Isabel I, Coatzacoalcos, Veracruz 96538, Mexico

## Abstract

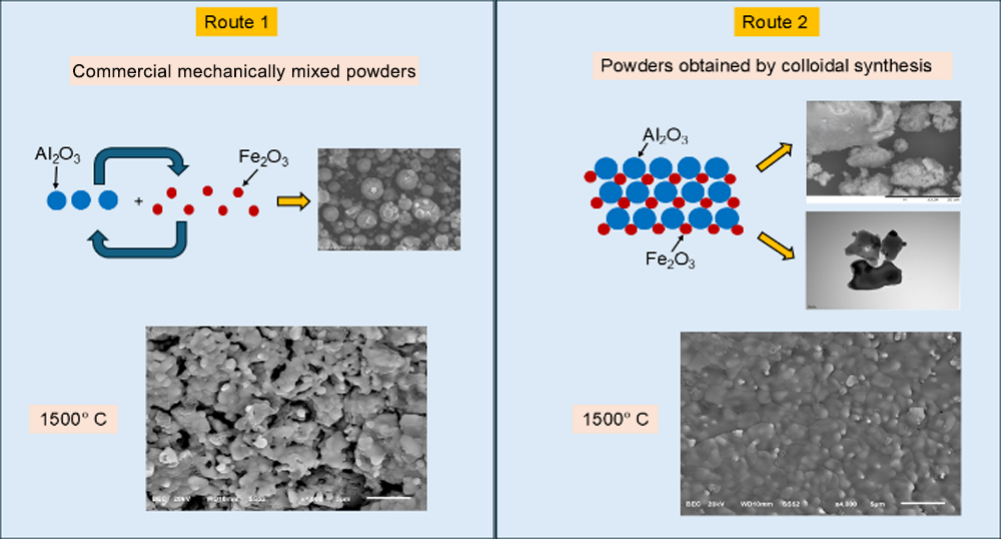

Morphological and microstructural changes induced by colloidal and traditional powder synthesis routes of nano-Al_2_O_3_–Fe_2_O_3_ mixtures were analyzed. Al_2_O_3_ was progressively replaced by 1, 2.5, 5, 10, and 20 wt % Fe_2_O_3_ nanoparticles. The replacement effect on dense ceramic composites formed by 100 MPa uniaxial pressure and sintered at 1500 °C was evaluated by transmission electron microscopy, scanning electron microscopy, and X-ray diffraction. After sintering treatment, the solid solution hercynite spinel-FeAl_2_O_4_ is obtained through a colloidal and a solid-state route (in all compositions). The utilization of the colloidal processing route allowed a homogeneous distribution of the Fe_2_O_3_ nanoparticles into the alumina matrix. Fe_2_O_3_ nanoparticles diffused at the Al_2_O_3_ grain boundaries, promoting better densification via hercynite formation. Also, the presence of Fe_2_O_3_ nanoparticles at grain boundaries acts, to a certain extent, as a pining component, promoting grain refinement. The colloidal synthesis route is a suitable alternative to promote densification in Al_2_O_3_–Fe_2_O_3_ dense ceramic composites.

## 1 Introduction

Alumina-based ceramics (Al_2_O_3_) have been extensively studied for structural applications due to their outstanding properties, such as dimensional stability (compressive strength, 2550–3100 MPa), resistance to high temperatures, and notable hardness (Vickers hardness, 20.6–29.4 GPa).^1−5^ Al_2_O_3_ exhibits traits like other polycrystalline ceramics, including moderate tensile (ultimate strength, 282 MPa) and bending (flexural strength, 152–800 MPa) strength and a tendency for brittle fracture (toughtness-K_IC_, 3.3–5 MPa·m^0.5^), which is a primary drawback of the mechanical performance of alumina. Al_2_O_3_ has low electrical (resistivity, 2 × 10^17^ Ohm·cm) and thermal (12.0–38.5 W/(m(K)) conductivities and an elevated melting point (T_M_, 2054 °C), which makes casting nearly impossible, and high hardness (9 Mohs), which renders machining challenging and costly.^4−6^

The brittleness of Al_2_O_3_ presents an additional concern in engineering design as it lacks the capacity for plastic deformation under stress. Al_2_O_3_ components are prone to fracture under high tensile stresses at points of surface imperfections, notches, and internal defects or when subjected to thermal shocks.^7^ Therefore, advancements in materials science and engineering have focused on the development of new techniques and materials, aimed at enhancing Al_2_O_3_ properties and mitigating disadvantages, thereby expanding their applicability as both structural and functional materials.^8,9^

One of the most common alternatives to enhance the properties of ceramics and mitigate their disadvantages is to add sintering additives with a low melting point, generating a liquid phase that allows for sintering and bonding of ceramic particles at reduced temperatures.^10^ This liquid phase created by additives “wets” the ceramic particles and flows between them, promoting particle rearrangement and enhancing densification. The surface tension of the liquid phase also aids in densifying the material and reducing porosity.^11,12^ Additionally, processing ceramics with the addition of two or more oxides at elevated temperatures leads to the formation of new crystal structures with unique properties, particularly beneficial to enhancing microstructural aspects of the original oxides.^13^

The Al_2_O_3_–Fe_2_O_3_ (alumina–hematite) compound, a mixed oxide system, has been widely investigated due to its physicochemical characteristics and is often used as a refractory material, sorbent, and catalyst component.^14−17^

Both alumina and hematite play roles in catalysis and adsorption.^17,18^ Key properties of alumina powders include surface acidity, mechanical robustness, high surface area, sintering stability, insulating properties, and diamagnetism.^4^ Meanwhile, hematite powders feature redox reactions, a moderate to low surface area, ease of sintering, semiconducting behavior, and antiferromagnetism due to the presence of iron.^19^ Hypothetically, combining Fe_2_O_3_ with Al_2_O_3_ could be advantageous, enabling the production of oxidation catalysts with enhanced stability or mechanically and thermally robust powders with redox capabilities. This is particularly true in industries that seek to reduce pollutant emissions in the production of chemical products, such as wastewater purification,^20^ selective oxidation reactions,^21^ and clean combustion processes,^22^ among others.

Some factors that influence the percentage of hematite (5–30% Fe_2_O_3_) in alumina^23^ include the type of oxidation reaction, the structure of the Al_2_O_3_ support, and operating temperatures. This percentage is adjusted to balance efficiency and cost when looking for stable and long-lasting catalysts for large-scale applications.

Furthermore, the Al_2_O_3_–Fe_2_O_3_ system has unique attributes^4,13,15−19,24−29^; however, concerning alumina properties when hematite is used as a sintering aid, only Wang et al.^29^ have investigated ceramic densification and flexural strength with varying Fe_2_O_3_ contents.

As a refractory material, alumina (Al_2_O_3_) is one of the most widely used products for preparing refractory materials and industrial furnaces. High-alumina brick (≤3% Fe_2_O_3_) is used in the safety layer of the electric arc furnace, the roof of the electric arc furnace, the safe area of the rotary cement, and in places with high abrasion resistance, since its service temperature goes up to 1800 °C.^30^ Currently, obtaining undesired porosity in Al_2_O_3_–Fe_2_O_3_ refractory bricks is a problem since inefficient or poorly controlled mixing can result in issues such as heterogeneous dispersion, resulting in rich zones of some components and incomplete interaction between particles during sintering. Thus, the chemical reactions generated at high temperatures are not always uniform and can cause the appearance of zones of uneven shrinkage due to the generation of internal stress caused by differences in thermal expansion coefficients or simply by the volume of the second phases generated.

On the other hand, control over the final microstructure of a sintered component requires an intimate connection between the initial material and the processing pathway inherent in the manufacturing method.^31^ In this sense, a traditional method used to consolidate high-quality ceramics is colloidal synthesis, which can produce dense, complex-shaped components with engineered microstructures.^31,32^ Colloidal processing methods are techniques that can control and improve the properties of the final compact through an excellent dispersion that produces a green compact with a narrow pore size distribution.^33−35^ Indeed, colloidal methods enable the achievement of high microstructural homogeneity in green and sintered parts and offer near-net shaping capabilities that reduce postsintering machining and production costs, which usually cannot be fully realized in more traditional particle processing.^35−38^

The origins of colloidal processing of ceramics are found in the ancient manufacture of earthenware objects, taking advantage of the plasticity of clays. Clay particles dispersed in water constituted the first colloidal dispersions prepared and used by humans, but always obeying experimental practice without a real understanding of the processes involved.^39,40^ The colloidal processing technologies for ceramics are usually based on the control of forces between particles in suspensions. By controlling the dispersion and stabilization of ceramic particles in a specific solvent or suspension, the agglomerates between ceramic particles can be broken down, and an intimate mixing degree between different compounds can be obtained, which can minimize the possibility of defects in the final material.^41−44^ In colloidal systems, the particles remain dispersed during the preparation of the suspension and during the consolidation step. In the absence of agglomerates, the consolidated green body should preserve the dispersion state of the colloidal suspension, thus resulting in the desired microstructural uniformity. A distinguishing feature of all colloidal systems is that the contact area between particles and the dispersing medium is large. As a result, interparticle (or surface) forces strongly influence the suspension behavior. Colloidal processing offers the potential to reliably produce ceramic films and bulk forms through careful control of the initial suspension “structure” and its evolution during fabrication.^37,38,44−46^

Colloidal nanomaterials, where the morphology, size, and phase composition are influenced not only by synthesis conditions but also by factors such as reagent and solvent purity, are in high demand for applications in optoelectronics, catalysis, biological imaging, ceramic, and sensing materials. In recent years, the colloidal method has emerged as the dominant synthetic protocol for producing nanomaterials, such as metals and metal chalcogenides, offering precise control over the nanoparticle size, composition, and morphology, key parameters in advancing materials science.^47,48^ Recently, researchers have used colloidal synthesis in combination with new sintering techniques, such as spark plasma sintering, mainly to distribute the second phase on the ceramic matrix.^49−53^

Regarding the Al_2_O_3_–Fe_2_O_3_ (alumina–hematite) system created from colloidal boehmite seeded with hematite particles or Fe(NO_3_)_3_ solution, McArdle and Messing^54^ found that the ferric nitrate solution shows a significantly higher nucleation efficiency compared to particulate seeds. They attributed this to the greater oxide particle concentration achieved through in situ ferric oxide formation, resulting in a higher nucleation frequency and enhanced interfacial contact between seeds and the matrix. This interpretation aligns with findings from other researchers^55^ and reinforces the crystallographic seeding effect discussed previously. Coprecipitated alumina–ferric oxide phases from alkoxide and Fe(NO_3_)_3_ solutions^56^ or Al and Fe nitrates^57^ displayed distinct phase transformations. In both solution routes, the first crystalline phase was γ-(Al, Fe)_2_O_3_ with a spinel structure. Polli et al.^57^ proposed that γ-(Al, Fe)_2_O_3_ directly converts to α-(Al, Fe)_2_O_3_ without the need for prior nucleation of ferric oxide seeds. Bye and Simpkin^56^ noted that Fe-rich cluster segregation within the γ-(Al, Fe)_2_O_3_ matrix may enhance α-Al_2_O_3_ formation by facilitating nucleation at lower temperatures and acting as seeds for α-Al_2_O_3_. These two distinct effects of Fe^3+^ ions on α-Al_2_O_3_ crystallization diverge from the epitaxial–crystallographic effect and were previously categorized by McArdle and Messing^54^ as a solution seeding effect.

This work highlights the importance of colloidal synthesis in refractory brick production and catalysts for exhaust gas treatment applications. In the literature, no studies have described a morphological and microstructural analysis through colloidal synthesis followed by thermal treatment for possible use in these applications.

Based on the above, this study seeks to broaden the understanding of nano-Al_2_O_3_–Fe_2_O_3_ mixtures obtained by two processing routes, (i) traditional powder mixture and (ii) colloidal processing route, by examining their behavior after sintering in terms of morphology and microstructural characteristics.

## 2 Experimental Procedure

### 2.1 Raw Materials

Two processing routes were employed to obtain the Al_2_O_3_–Fe_2_O_3_ composites: mechanical mixing of powders (route 1) and colloidal synthesis (route 2). The raw materials used in route 1 consisted of analytical-grade powders of aluminum oxide (Al_2_O_3_, 99.9% purity, with a size of ≈150 nm) and hematite or iron(III) oxide (α-Fe_2_O_3_, 99% purity with a particle size of 20–40 nm) both supplied by J.T. Baker. In the case of route 2, the raw materials consisted of aluminum oxide (Al_2_O_3_, 99.9% purity) and iron(III) nitrate nonahydrate (Fe(NO_3_)_3_·9H_2_O) supplied by Fermont as the precursor of iron(III) oxide. The compositions used for both routes were (100 – *X*) wt % Al_2_O_3_ + *X* wt % Fe_2_O_3_, where *X* = 0, 1, 2.5, 5, 10, and 20. As is known, the Al_2_O_3_–Fe_2_O_3_ system is used for various purposes. Specifically, it is employed in the development of refractory materials (0–3 wt % Fe_2_O_3_)^24−29^ and catalysts (1–30 wt % Fe_2_O_3_).^23^ We added up to 20 wt % Fe_2_O_3_ to account for compositions used in both applications. Percentages above 20 wt % Fe_2_O_3_ would significantly increase costs for catalytic purposes, and an excess of Fe_2_O_3_ (>20 wt %) could also negatively impact the thermal stability of the catalyst.

On the other hand, in refractory materials, an excess of Fe_2_O_3_ (>20 wt %) can microstructurally degrade the matrix, as an oversaturation of Fe_2_O_3_ grains within the alumina matrix may compromise thermal stability. Specifically, when the brick is exposed to high temperatures, low-melting-point phases can form negatively, impacting the microstructure and thermal stability of the refractory brick.

The use of nanoparticles as starting materials in a homogeneously distributed state offers several advantages in producing advanced materials. In this study, nanoparticles were used to achieve sintered grains on the micrometer scale, resulting in densified pieces. Furthermore, it has been shown that due to their high surface area, nanoparticles require less energy for sintering, allowing a sintering temperature reduction. The final sintered piece can attain a homogeneous microstructure with excellent physical, mechanical, and thermal properties suitable for advanced applications.^58^

### 2.2 Sample Preparation of Al_2_O_3_ Alumina/Fe_2_O_3_ Hematite Composites

**Figure 1:**
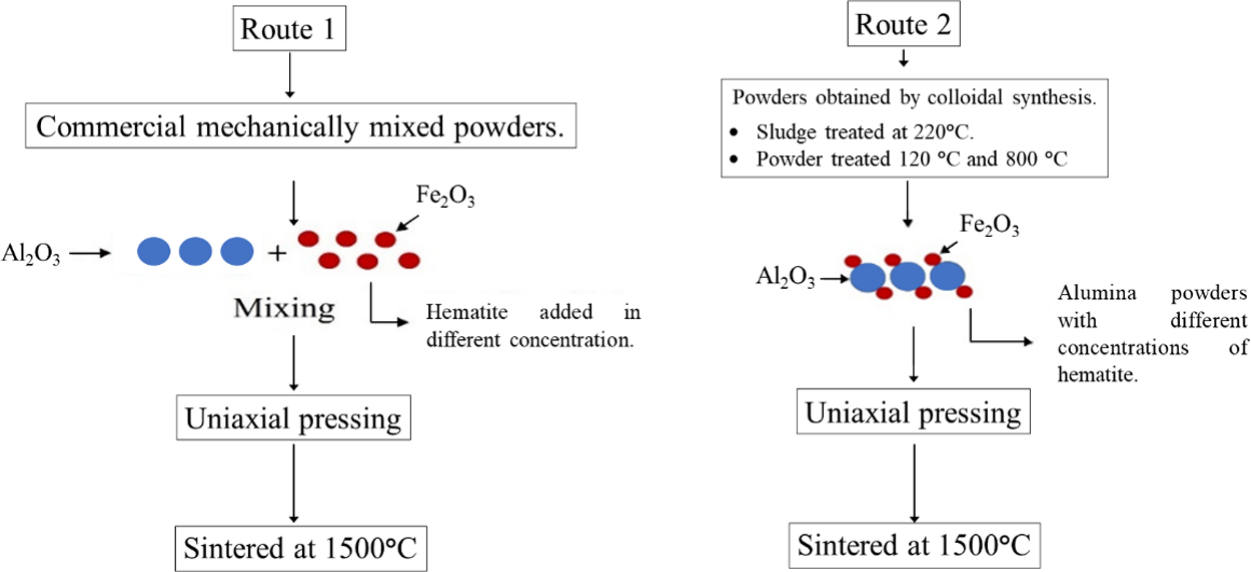
Flow diagram of the preparation process of the samples by **Route 1** (R1) and **Route 2** (R2).

Figure 1 shows a flow diagram of the two routes (R1 and R2) proposed to elaborate on the ceramic composites.

#### 2.2.1 Route 1

To obtain a suitable and homogeneous mixture of alumina–hematite powders, a dry mechanical mixing process was carried out by using a mechanical mixer (Alghamix II-Zhermack) for 15 min at 100 rpm. After that, the powder was poured into a steel mold to perform uniaxial pressing at 100 MPa. Refractory samples with a diameter of 12 mm and a height of 5 mm were obtained. Finally, the refractory samples were sintered in an electric furnace at 1500 °C (with a mildly reducing atmosphere) at a rate of 5 °C/min for 4 h.

#### 2.2.2 Route 2

First, alumina and iron(III) nitrate nonahydrate were dissolved in water separately. The iron nitrate nonahydrate was dissolved in water by heating to 100 °C and 80 rpm. Then, the nitrate solution was poured drop by drop onto the alumina solution. At the same time, under magnetic stirring, the suspension was heated to 220 °C at 120 rpm. The mud was subsequently heated in a muffle furnace to 120 °C for 24 h in an air atmosphere to start the nucleation of the composite and to remove the water. Dried powders were sieved at 180 μm. Subsequently, these powders were treated at 800 °C for 4 h in an air atmosphere to remove nitrates and reduce them. Treated powders were sieved under 180 μm. Subsequently, the powders were treated as in route 1, uniaxially pressed, and sintered at 1500 °C using a conventional furnace in a mildly reducing atmosphere.

### 2.3 Characterization

The crystal structure and identification of phases were carried out by the X-ray diffraction (XRD) technique (Bruker D8 Advance model) with CuK_α_ radiation (λ = 1.5406 Å), operated at 40 kV and 30 mA. Scans were performed in the 2θ range of 10–90° with a step scan of 0.05° and 1.5 s per step in a continuous mode. The powders obtained by colloidal synthesis and heated to 800 °C were characterized in a transmission electron microscope (TEM-JEOL 1011). The morphological analysis was performed by using an FEI Nova NanoSEM 200 scanning electron microscope equipped with an electron-dispersive X-ray spectroscopy (EDX) detector (EDAX, Apollo XP model, 2930 serial number).

## 3 Results and Discussion

### 3.1 XRD Analysis

**Figure 2:**
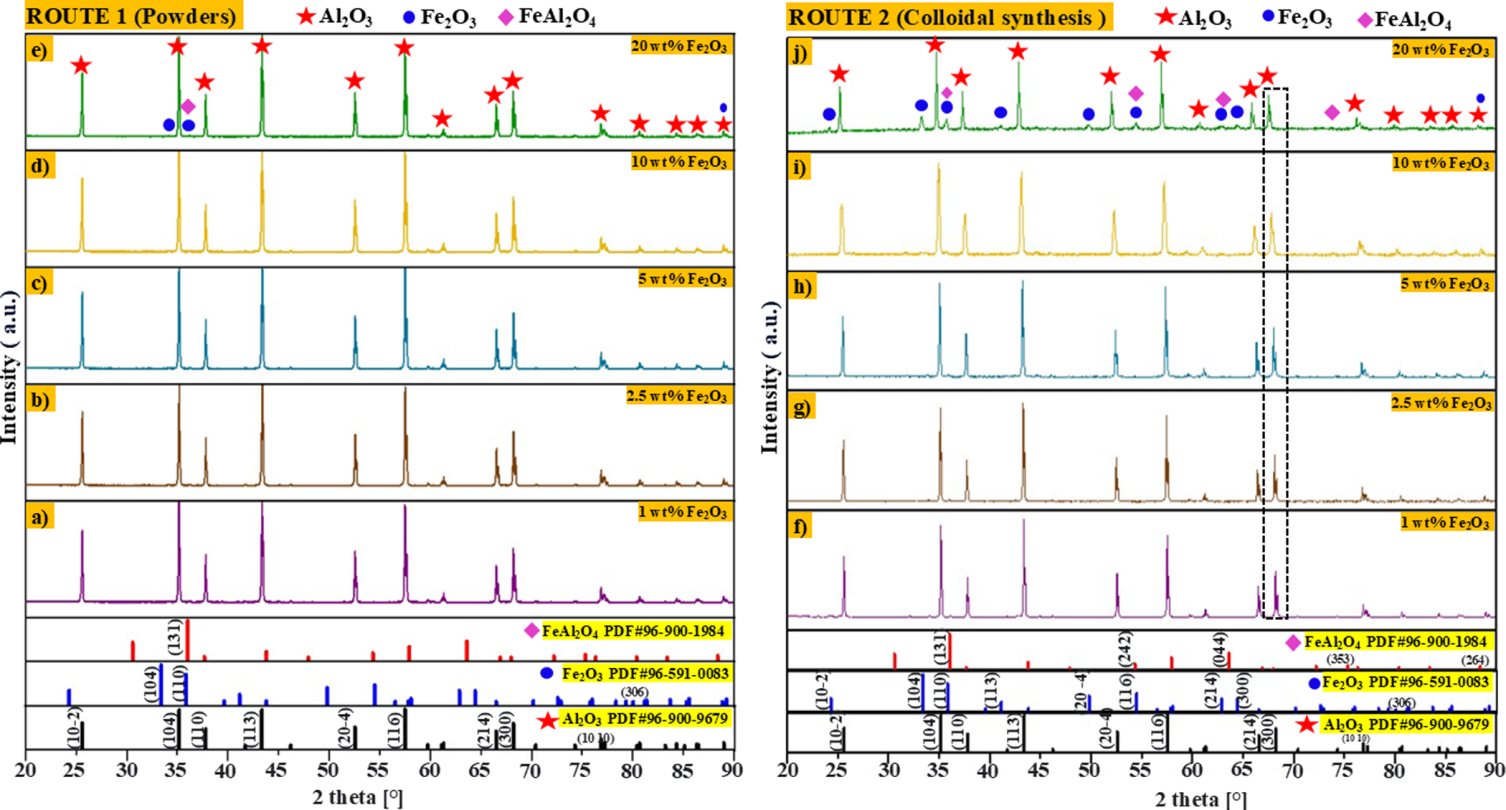
XRD patterns of raw materials and sintered samples performed by routes 1 and 2.

Microstructural analysis of sintered samples at 1500 °C for both routes 1 and 2 is given in Figure 2a–j. Figure 2a–e shows the XRD patterns of route 1 with concentrations of 1, 2.5, 5, 10, and 20 wt % of Fe_2_O_3_ incorporated into the Al_2_O_3_ matrix, respectively. In Figure 2e, corresponding to the 20 wt % Fe_2_O_3_ sample, characteristic peaks of alumina are observed, which were identified with the PDF chart 96-900-9679 with reflections specifically at 2θ = 25.56°, 35.13°, 37.76°, 43.33°, 52.53°, 57.48°, 66.48°, 68.17°, and 76.85°, which correspond to the (10–2), (104), (110), (113), (20–4), (116), (214), (300), and (1010) planes of hexagonal Al_2_O_3_, respectively. Likewise, peaks belonging to iron oxide were observed at 2θ = 33.38° and 35.8° with reflections in the (104) and (110) planes of hexagonal hematite (Fe_2_O_3_), respectively (the standard pattern PDF 96-591-0083). A new phase was detected in the low proportion corresponding to hercynite at 2θ = 36.24° with a diffracted plane in (131) corresponding to a cubic structure of FeAl_2_O_4_, with the standard pattern PDF 96-900-1984. Microstructural analyses of the samples with 1, 2.5, 5, 10, and 20 wt % Fe_2_O_3_ incorporated into the Al_2_O_3_ matrix, respectively, by colloidal synthesis (route 2) were performed (see Figure 2f–j). When comparing both routes, a variation in peak intensities was noticeable, with higher intensities corresponding to route 2. When the colloidal synthesis powders were heated at 800 °C (before the pressing and sintering stages), iron nitrate underwent thermal decomposition, causing a gradual elimination of the nitrates and transforming them into Fe_2_O_3_. Later, when the samples were sintered at 1500 °C, a reaction occurred between alumina (Al_2_O_3_) and iron oxide, leading to hercynite formation. At 1 wt % Fe_2_O_3_, characteristic peaks of hercynite were detected. These peaks intensified as the Fe_2_O_3_ content was increased, leading to the detection of four new peaks at 2θ = 36.24°, 54.70, 64.08°, and 75.90° with reflections in the (131), (242), (044), and (353) planes, respectively, corresponding to hercynite with 20 wt % Fe_2_O_3_.

At high temperature and considering the arrangement of the Fe_2_O_3_ nanoparticles anchored on the surface of the alumina particles (see Figure 6d), surface and mass sintering mechanisms were carried out, facilitating the cationic migration of the 3+ valence of both materials. In this sense, a substitution of atoms from Al^3+^ with an ionic radius (0.53 Å) was occupied by Fe^3+^ atoms with an ionic radius of (0.6 Å), so a new phase was formed (hercynite). In this way, the sintering process is carried out in the solid state (Fe_2_O_3_ = 1535 °C and Al_2_O_3_= 2054 °C) by diffusion of the cation via vacancies.

The fact that Al ions, which were originally in a hexagonal crystalline structure of Al_2_O_3_, were replaced by Fe ions (hexagonal structure) mainly caused two basic sintering mechanisms within the material: the first is sintering by vacancies of alumina cations within the hexagonal structure of aluminum oxide: that is, due to crystalline defects or the nature of the material, there are cation vacancies within the bulk alumina or close to the periphery of the grain boundary of the aluminum oxide, which can be replaced at high temperature by Al_2_O_3_ or Fe_2_O_3_ cations that could be found either in the internal part of the grain (Al_2_O_3_) or in the external part of the alumina particles. Therefore, a migration toward the grain boundaries takes place, causing the grain boundaries to begin to move (grow) due to cationic movement, which moves in the same direction. The second mechanism is contraction of the material: this contraction causes the pores to close, since the boundary of one grain and another grow until they join, densifying the piece. This assumption is complemented by the micrographs shown in Figure 4, where samples with porosities and cracks are observed through route 1, while a densified morphology was observed with samples via route 2.

The chemical formula of hercynite is FeAl_2_O_4_, and it has a spinel-type structure.^59−61^ Its divalent ions prefer tetrahedral sites, while the trivalent ones occupy octahedral sites.^60^

Hercynite, as a refractory phase, has a hardness of 7.5 on the Mohs scale and melts above 1780 °C. Hercynite is used in refractory applications, hydrogen production,^62^ cement rotary kilns,^63^ and metallurgical furnaces, which are characterized by their thermal stability and corrosion resistance.^64^ It is also employed in catalysts as an active component, meaning that it can accelerate chemical reactions without being consumed, for instance, in hydrodesulfurization catalysts,^65^ mild hydrogenation catalysts,^62^ and oxidation catalysts.^66^

Although natural deposits of hercynite are scarce, it can be synthesized from its oxides through solid-state reactions or electrofusion.^67,68^ Hercynite synthesis takes place in arc furnaces at temperatures above 1500 °C. Various researchers have produced hercynite spinel (FeAl_2_O_4_) using alumina, hematite, and a reducing agent.^69^ Furthermore, hercynite formation (FeAl_2_O_4_) can be achieved under specific conditions. At 1500 °C, the oxide stability allows Fe_2_O_3_ to reduce and react with Al_2_O_3_, leading to the following process:

Partial dissociation of Fe_2_O_3_ can occur at high temperatures, according to the following reaction^70,71^:
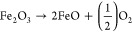
1

The amount of oxygen released depends on the atmosphere and the partial oxygen pressure. In this case, a mildly reducing atmosphere was used. Once FeO is present, it can react with alumina (Al_2_O_3_) to form hercynite at 1500 °C. This chemical principle is a solid-state reaction, producing a stable phase at high temperatures.
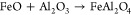
2

Likewise, if conditions are not controlled (e.g., hematite content exceeds 10 wt % Fe_2_O_3_ or there is a significant amount of oxygen in the atmosphere), residual hematite and unreacted alumina will be detected.

Thermodynamic calculations for the synthesis of hercynite (FeAl_2_O_4_) were performed using HSC5.1 software to determine whether the reaction product is thermodynamically favorable under the parameters used in this study. As shown in Table 1, Gibbs’s free energy values are negative for all simulated temperature cases, indicating that the reaction product is spontaneous and can occur at the tested temperatures presented in Table 1. In this study, 1500 °C was used as the synthesis temperature in response to the typical sintering temperature of alumina, facilitating the densification of the material. Table 1 also provides the reaction enthalpy, which is the minimum energy required for reaction spontaneity. The reaction enthalpy was achieved using a controlled temperature inside the furnace. These values validate the experimental results.

**Table 1:** Thermodynamic Values for the Synthesis of FeAl_2_O_4_

thermodynamic values for the synthesis of FeAl_2_O_4_					
*T* (°C)	Δ*H* (kJ)	Δ*S* (J/K)	Δ*G* (kJ)	*K*	log(*K*)
1200	–62.819	–15.466	–40.035	2.63 × 10^01^	1.42
1300	–63.468	–15.893	–38.466	1.89 × 10^01^	1.277
1400	–88.153	–30.86	–36.52	1.38 × 10^01^	1.14
1500	–88.918	–31.304	–33.411	9.65 × 10^00^	0.984
1600	–89.499	–31.624	–30.263	6.98 × 10^00^	0.844

**Figure 3:**
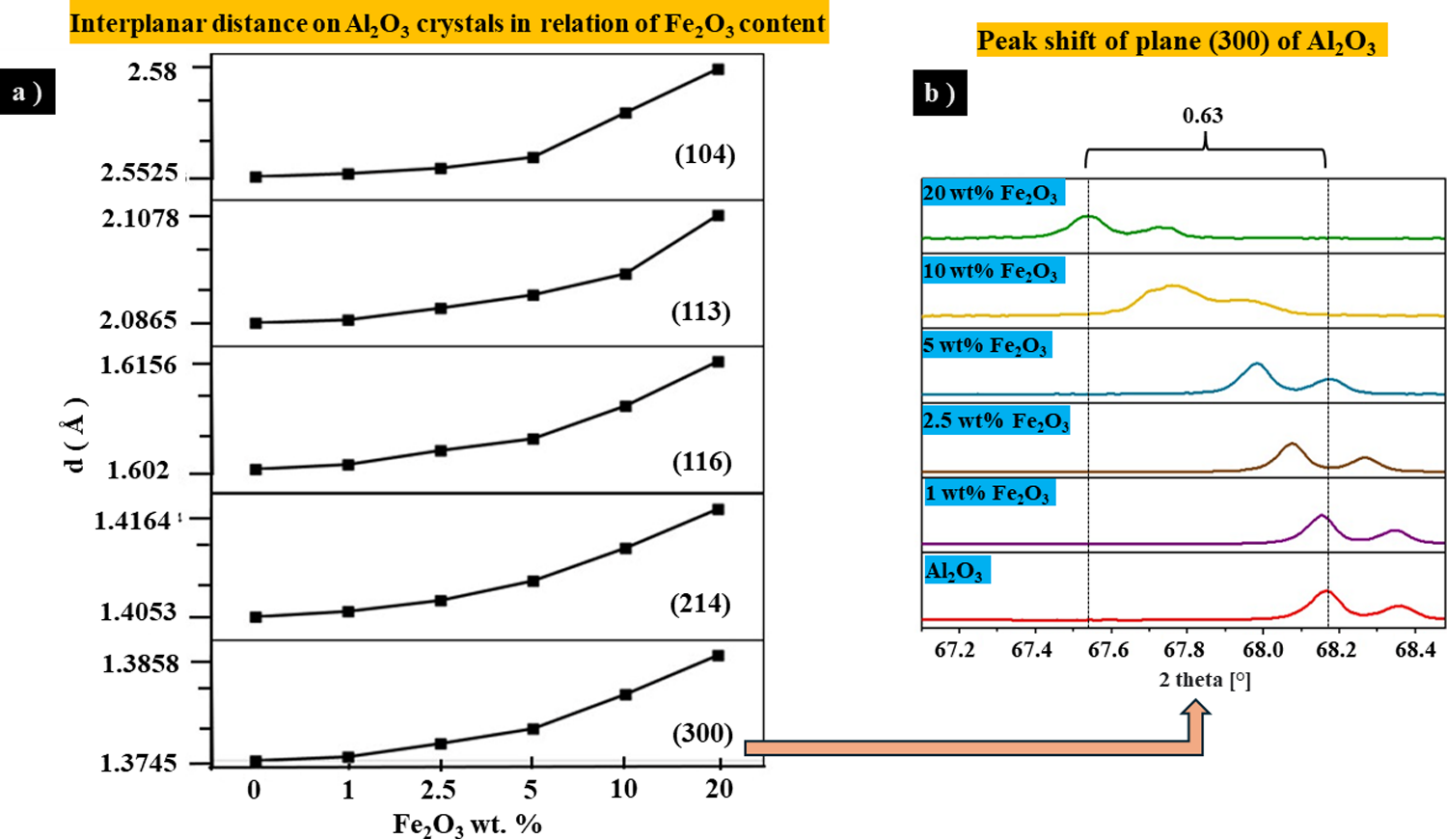
(a) Values of the interplanar distance “*d*” on Al_2_O_3_ crystals in relation to the Fe_2_O_3_ content for the five peaks with the highest relative intensity. (b) Shift of peaks about the alumina peak at 68.18° depending on the concentration of Fe_2_O_3_.

In Figure 2f–j, a peak shift is observed. This can be explained as follows: when atoms of iron oxide (Fe_2_O_3_) entered the aluminum oxide lattice (Al_2_O_3_), it caused a crystalline distortion of the hexagonal crystal lattice of Al_2_O_3_. This phenomenon was observed due to the shifts of the alumina peaks (see dotted line in Figure 2f–j) toward smaller angles 2(θ) depending on the concentrations of Fe_2_O_3_ by route 2. This shift to the left is mainly assigned to (i) the larger atoms’ replacement within crystalline structures with smaller atoms^72^ or (ii) the excessive concentrations of a dopant within the matrix. Likewise, in Figure 3a, it is observed that the interplanar distance of the principal planes of alumina (104), (113), (116), (214), and (300) increased corresponding to the concentration (wt %) of Fe_2_O_3_. The most evident displacement 2(θ) was 0.63°, corresponding to the difference in angles of 68.18° for alumina (Al_2_O_3_) and 67.55° for Al_2_O_3_ + 20 wt % Fe_2_O_3_ from the (300) plane (see Figure 3b).

### 3.2 Morphological Analysis (SEM–EDX Analysis)

**Figure 4:**
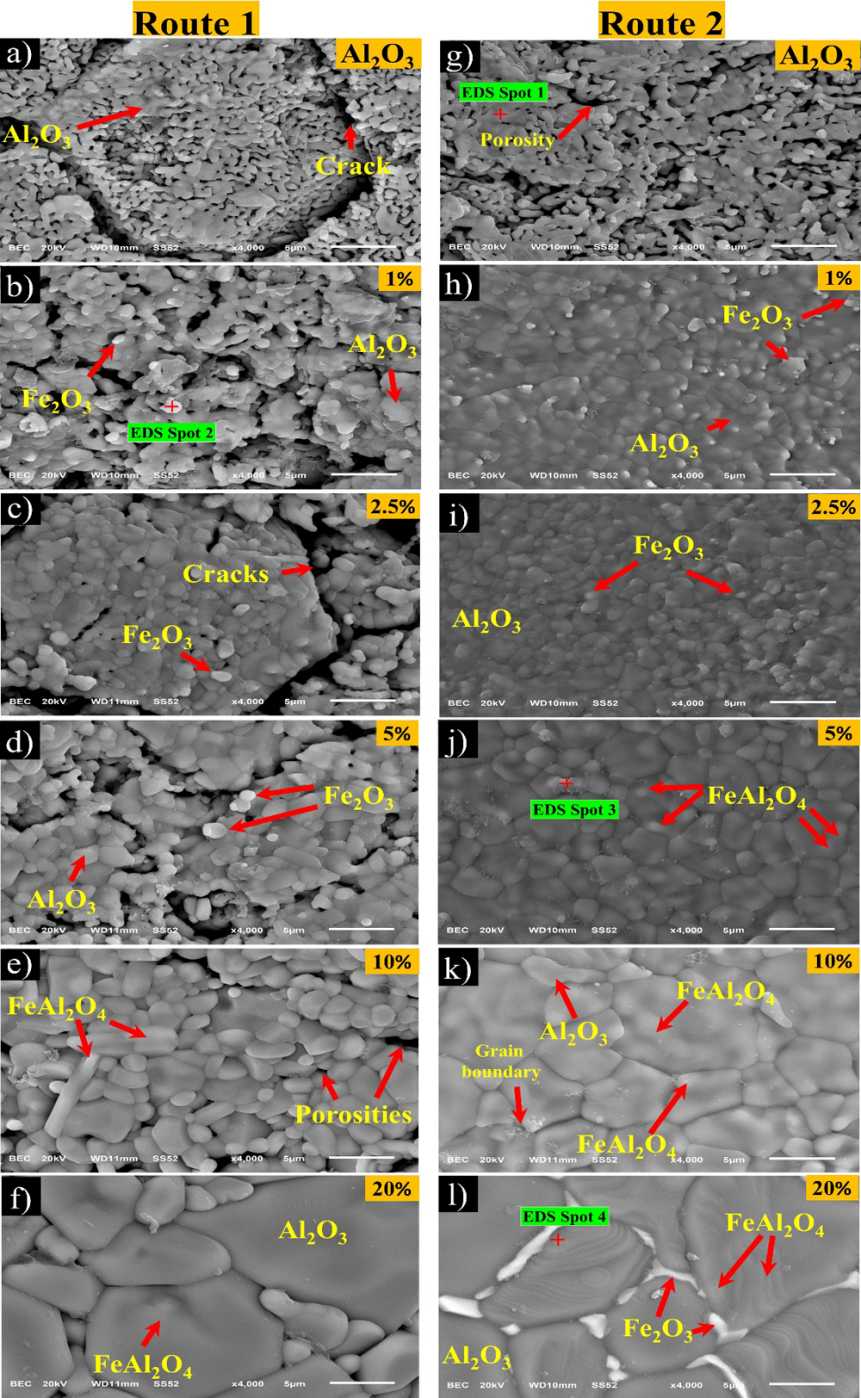
SEM micrograph of alumina processed by both routes (1 and 2) with different concentrations of Fe_2_O_3_ (a) and (g) without additions of Fe_2_O_3_, (b) and (h), (c) and (i), (d) and (j), (e) and (k), (f) and (l) corresponding to 1, 2.5, 5, 10, and 20 wt % Fe_2_O_3_, respectively. All of the samples were sintered at 1500 °C.

Figure 4 shows scanning electron microscopy (SEM) images of samples processed by routes 1 and 2. Figure 4a–f corresponds to images of samples with a mixture of analytical-grade powders (route 1). Figure 4a shows the refractory matrix (Al_2_O_3_) composed of particles with a size of ∼1 μm, open porosities, and cracks. At 1 and 2.5 wt % Fe_2_O_3_, hematite particles dispersed on Al_2_O_3_ particles are observed. From here, it is evident that the Al_2_O_3_ particle size increased as Fe_2_O_3_ was added. Besides, the formation of cracks and porosities was also perceptible (see Figure 4b,c). At 5 wt % Fe_2_O_3_, the Al_2_O_3_ particle growth was observed in conjunction with porosity and cracks between Al_2_O_3_ particles (see Figure 4d). At 10 wt % Fe_2_O_3_, it was clear that the hematite was semidistributed on the Al_2_O_3_ grain, causing Al_2_O_3_ growth grain between 5 and 10 μm in size (see Figure 4e). At 20 wt % Fe_2_O_3_, abnormal and heterogeneous grain growth (∼20 μm) of Al_2_O_3_ was observed (see Figure 4f). Also, bright white areas at Al_2_O_3_ grain boundaries were observed, which correspond to hematite and the FeAl_2_O_4_ phase.

Figure 4g–l corresponds to images of samples processed by colloidal synthesis (route 2). In Figure 4g, corresponding to Al_2_O_3_ powders, the formation of necks between grains with porosity distributed on the sample was observed. At 1 and 2.5 wt % Fe_2_O_3_, a substantial improvement in the morphology of both samples was evident since densified, compact, and homogeneous grains (∼1–3 μm) without porosity (porosity free) or cracks were observed (see Figure 4h,i). At 5 wt % Fe_2_O_3_, the increase and homogeneity in Al_2_O_3_ grain size were observed (∼5 μm); at 10 wt % Fe_2_O_3_, the Al_2_O_3_ grain size increased at ∼10 μm (see Figure 4j,k, respectively). At 20 wt % Fe_2_O_3_, the grain size increased at >10 μm size. However, Fe_2_O_3_ supersaturation in the crystal structure of Al_2_O_3_ was evident. Hematite was detected in the grain limits as bright white areas (see Figure 4l).

Although no physical density tests were conducted, it was observed in Route 2 that the porosity significantly decreased in all presented micrographs (Figure 4h–l). When Al_2_O_3_ and Fe_2_O_3_ reacted, an initial structural reorganization occurred, occupying less space within the matrix and leading to a new phase formation, hercynite. This phase facilitated greater atomic diffusion during sintering, allowing atoms to reorganize easily to fill voids or pores, ultimately producing a denser and more complex structure. Furthermore, the intrinsic density of the formed hercynite is higher (4.6 g/cm^3^) than that of the matrix (alumina, 3.9 g/cm^3^). The formation of hercynite within the matrix, being a denser phase, contributes to an overall increase in the global density of the composite.

**Figure 5:**
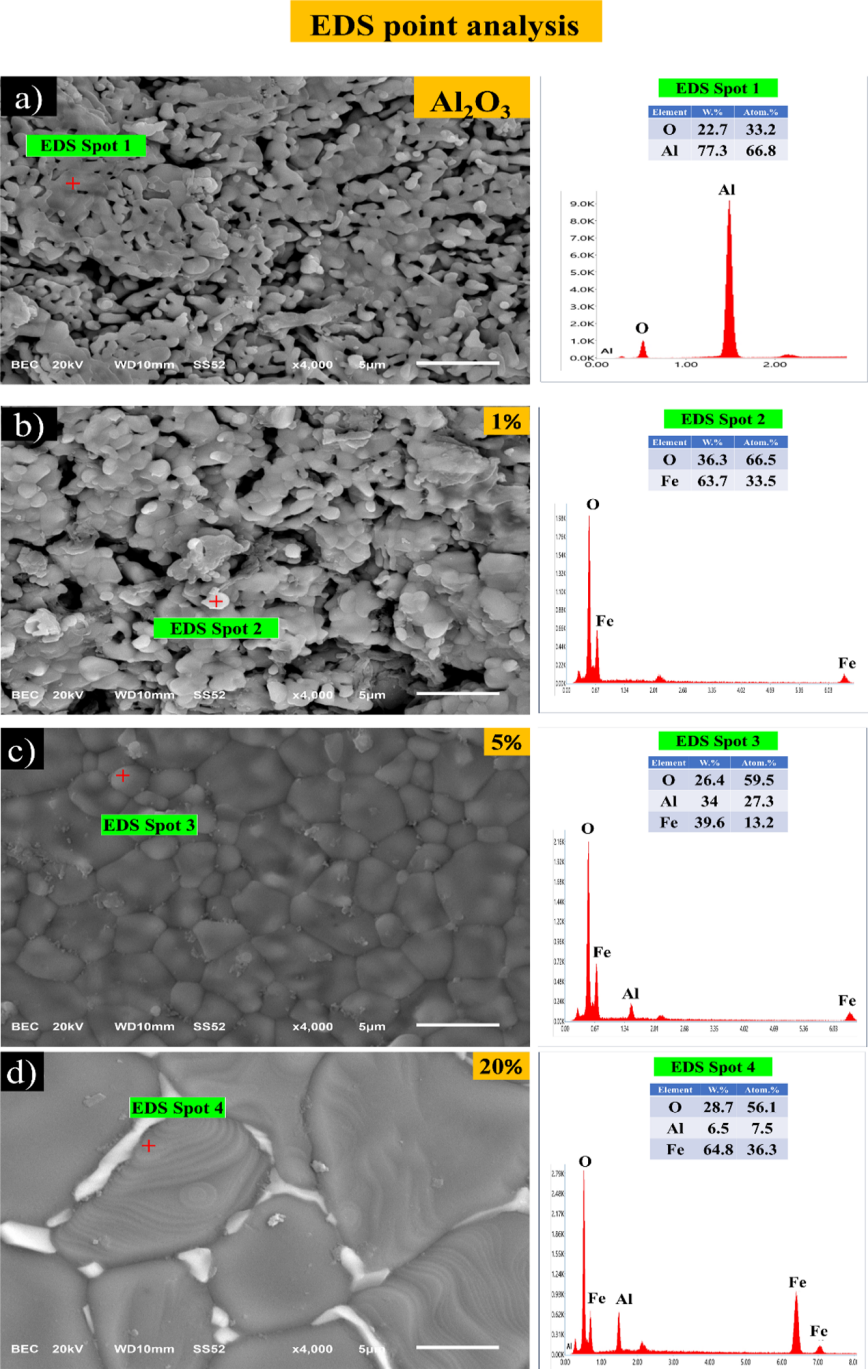
Spot EDS analysis of samples processed by both routes (1 and 2) of alumina with different concentrations of Fe_2_O_3,_ (a) Al_2_O_3_ sample by route 2 (without the addition of Fe_2_O_3_), (b) 1 wt % Fe_2_O_3_ (by route 1), (c) and (d) corresponding to 5 and 20 wt % Fe_2_O_3_, respectively (both by route 2). All of the samples were sintered at 1500 °C.

An EDS spot analysis was conducted on four different samples. In these analyses, alumina was identified as the dark gray phase (spot 1, Figure 5a), while the bright white round spots corresponded to hematite (spot 2, Figure 5b). Within the alumina grains, a whitish-gray phase was identified as hercynite (spot 3, Figure 5c). In addition, inside the alumina grains (spot 4, Figure 5d) with the 20 wt % Fe_2_O_3_ sample, a greater amount of Fe with Al was detected, which can be attributed to hercynite concentrations.

The differences in microstructure characteristics between the two routes are closely linked to the dispersion methods used in routes 1 and 2.

In this context, the pinning effect observed in route 2 depends on how the Fe_2_O_3_ particles are well-distributed and adhered within the Al_2_O_3_ matrix. In the colloidal synthesis process, material preparation allowed for a homogeneous dispersion and distribution of Fe_2_O_3_. This fact enables Fe_2_O_3_ nanoparticles to position themselves directly at grain boundaries, acting as physical barriers that limit grain boundary growth or movement, thus inhibiting diffusion mechanisms among alumina particles. In addition, in route 2, the Fe_2_O_3_ nanoparticles are more likely to chemically react with Al_2_O_3_, which also develops chemical pinning. This fact involves stable chemical interactions that enhance the Fe_2_O_3_ pinning effect via FeAl_2_O_4_ formation. FeAl_2_O_4_ is a rigid phase that counters the atomic movement among Al_2_O_3_ particles.

On the other hand, in route 1 (mechanical mixing), particles are generally dispersed in a less controlled and more heterogeneous manner with weaker adhesion to the matrix. This fact means that Fe_2_O_3_ particles tend to agglomerate or cluster together, making it difficult for them to position themselves at grain boundaries, resulting in a less effective or nonexistent pinning effect. Consequently, surface and bulk diffusion mechanisms are predominant, leading to more evident alumina grain growth than in route 2.

#### 3.2.1 Particle Sizes and Morphologies of Both Routes

**Figure 6:**
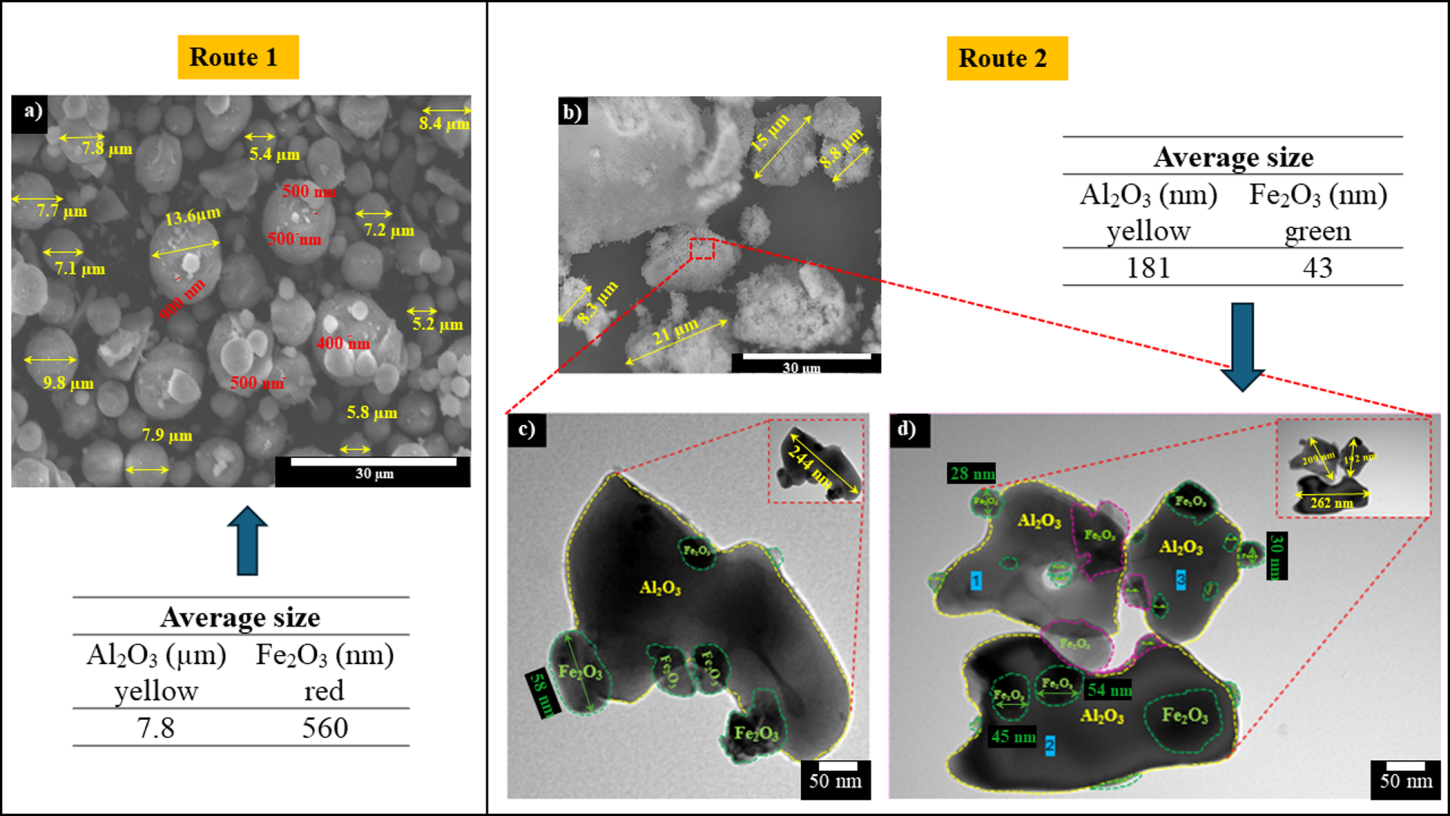
SEM micrographs of particles processed by (a) route 1 and (b) route 2 both with 5 wt % Fe_2_O_3_; (c and d) TEM images from route 2. In both routes, the average particle size of Al_2_O_3_ and Fe_2_O_3_ was calculated (inset tables). Only particles processed by route 2 were calcined at 800 °C.

Figure 6a displays SEM images of powders of commercial mechanically mixed powders (route 1). Figure 6b–d displays SEM and TEM images of powders obtained by colloidal synthesis after calcination at 800 °C (route 2). In both cases, 5 wt % Fe_2_O_3_ addition was considered.

In Figure 6a, larger Al_2_O_3_ particles are observed compared to route 2. These particles (alumina agglomerates) had an average size of 7.8 μm. Particle traces of about 560 nm corresponding to hematite (Fe_2_O_3_) were detected on Al_2_O_3_ particles (see the table on the left in Figure 6). The above observations indicate that although ideal parameters from other mechanical mixing experiments were used, in this case, dry mixing failed to disperse the particles, yielding particles with even micrometric sizes.

Figure 6b shows agglomerates formed after calcination. These agglomerates were formed by small nanometric Al_2_O_3_ particles surrounded by nanometric Fe_2_O_3_ particles. These clusters had an average size of 13.2 μm. Figure 6c,d displays TEM images of the particles that made up these clusters. Al_2_O_3_ nanoparticles have an average size of 181 nm. Al_2_O_3_ nanoparticles were surrounded (anchored) by hematite particles with an average size of 43 nm (see table on the right in the inset in Figure 6).

The effect of the colloidal synthesis causes homogeneous dispersion of iron nitrate particles through alumina particles. Subsequently, during the calcination stage, a rapid nucleation process occurs, forming a defined number of crystals of Fe_2_O_3_ at a nanometric scale. These nanoparticles, when superficially anchored (in a physical manner) to the skin of alumina particles (as seen in the particles outlined in purple in Figure 6d), with thermal treatment at high temperatures, can inhibit the different mass transport mechanisms between other alumina particles (see particles identified by numbers in blue (1, 2, and 3) in Figure 6d). This mechanism was enhanced at 1–5 wt % Fe_2_O_3_ via route 2, as seen in Figure 4h–j.

## 4 Conclusions

In this work, two comparative processing routes were performed to obtain densified alumina composites with different concentrations of Fe_2_O_3_. Route 1 (traditional mixing of powders) started with analytical-grade commercial powders. Meanwhile, route 2 (colloidal synthesis route) started with analytical-grade commercial alumina and iron(III) nitrate nonahydrate as the precursors of Fe_2_O_3_. The effect of the processing route on the morphological and microstructural characteristics of the Al_2_O_3_–Fe_2_O_3_ composites was studied for these two routes.

Microstructurally, the addition of hematite to the alumina matrix caused the formation of an in situ phase (FeAl_2_O_4_, hercynite), which occurred by reactive sintering. This phase appears in all of the compositions, as revealed in the SEM–EDX images, although it is more visible in the case of the composite with 20 wt % Fe_2_O_3_. Likewise, the hematite distorted within the alumina network, provoking a shift of peaks toward smaller angles 2(θ). This distortion is due to the formation of the newly detected phase.

Morphologically, the sintered specimens obtained by the colloidal synthesis exhibit a more densified appearance. Evident densification is observed during the microstructural analysis in the samples with 1, 2.5, and 5 wt % Fe_2_O_3_ obtained through route 2. A densified composite with homogeneous grains, particle sizes of 1–3 μm, porosity, and absence of cracks was obtained. Meanwhile, in all of the specimens sintered from the traditional powder method (route 1), porosity and crack formation were evident.

At the highest addition of Fe_2_O_3_ by route 1 (20 wt % Fe_2_O_3_), large grains with small porosity were observed. This grain growth in the case of route 1 means that hematite does not have a pinning effect on the grain growth. In contrast, in the composite obtained by the colloidal route, hematite appears to contour the grains of alumina, controlling grain growth and promoting densification. Therefore, the colloidal synthesis route emerges as a promising alternative to obtain dense Al_2_O_3_–Fe_2_O_3_ composites for use in applications that handle this binary system.
